# Hereditary Neuroses in Children.—V

**Published:** 1897-06-19

**Authors:** 


### Modern Sociology.

### HEREDITARY NEUROSES IN CHILDREN.—V.

The relation of inherited constitutional taints to neurotic conditions in early life is a subject of great interest, but one to which we can only briefly refer. We have already remarked the frequency of a phthisical family history in the case of mentally-deficient children; and in our last article we showed how inherited syphilis might produce a species of juvenile dementia or general paralysis. Clouston observed, as long ago as 1863, the hereditary relationship of insanity and phthisis, as, indeed, McKinnon, Van der Kolk, and Guislain had also done. Maudsley has argued that a morbid neurosis may manifest itself not only in disorder of sensation, motion, or mentality, but also in disorder of nutrition, whereof diabetes is the earlier, and phthisis the later, stage. We need not then be surprised to find that a phthisical family history is so preponderating a factor in the etiology of idiocy and imbecility (being found in 28·31 per cent. of the cases tabulated by Shuttleworth and Beach), or that two-thirds of the mortality of mentally-defective children is due to some form of tuberculous disease. But the public are not sufficiently alive to the risk of marriage into a consumptive family being productive not only of phthisical, but also of mental disease in the offspring; and it may be well to point out that medical experience has fully ascertained the existence of this risk. The blend of neurotic and phthisical heredity seems to be specially harmful: and a case might be cited in which a consumptive father and a nervous mother had a family of five, two of whom were idiotic, one a moral imbecile, and the other two (who were not mentally defective) developed pulmonary phthisis at adolescence.

Inherited syphilis produces, as we have seen, a specific mental and nervous degeneracy in the offspring, and this is one of the remote effects, which (as well as others more immediate) may well cause a man who has suffered from constitutional syphilis to pause ere he commits the crime of matrimony. If the percentage of cases of inherited syphilis in the records of idiot institutions is but small, this is probably in some measure due to the difficulty in such cases of obtaining accurate histories.

Alcoholism figures in asylum statistics as a prominent cause of insanity, but the mischief is not limited to one generation, and the transmitted effects show themselves in various forms of neurosis affecting the progeny. The matter is, however, not quite so simple as has been popularly supposed. The ancient adage, "*Et patrum in natos abeunt cum semine mores*," has been applied as if the effect of the drunken debauch of the parent necessarily imprints itself on the features, physical and mental, of the child engendered under such circumstances. The late Dr. Langdon-Down,* indeed, long ago adduced cases to show that the cretinous type of idiocy—a type associated with elfish form and shambling gait—was the direct product of drunken procreation. Recent discoveries as to the absence in such cases of defect in the development of the thyroid gland, with mental and physical arrest due to want of metabolism consequent on the absence of its secretion, do not confirm this view. Though no one will deny that drunkenness is the prolific parent of crime, disease, and poverty, it may perhaps be questioned whether idiocy in the offspring is its direct Nemesis in so large a percentage of cases as some would have us believe. American statistics, gathered fifty years ago under conditions hardly favourable to scientific accuracy, are still quoted to the effect that nearly one-half of the parents of idiotic children are intemperate. According to the combined investigations of Drs. Shuttleworth and Beach, previously referred to,* parental intemperance figures as a factor in no more than 16·38 per cent. of their 2,380 cases, special care having been taken in the compilation of their statistics on the subject. There are, however, other neuroses which the child of the drunkard inherits, such as deaf-mutism, a tendency to convulsions in infancy, to epilepsy in after-life, and to alcoholic craving, the latter sometimes showing itself very early in life. A pedigree set out by Morel in his "Traité des Dégénérescences" is very striking. In the first generation we find a grandfather who has permitted himself to become a habitual drunkard. In the second figures an inebriate son, who disgraced himself by disgusting drunkenness on his own marriage day. In the third come seven grandchildren, offspring of the last-named; of these the first and second died of convulsions in infancy, the third was an idiot, the fourth grew up a suicidal melancholiac, the fifth was of peculiar irritable disposition, the sixth was repeatedly insane, and the seventh nervous and depressed! Dr. Strahan† draws a distinction between the chronic habitual drunkard and the paroxysmal dipsomaniac, and opines that the form of inheritance from the former is apt to be idiocy, deaf-mutism, scrofula, and phthisis; whilst from the latter result the more acute manifestations of mania, suicide, and epilepsy. Early and severe neuralgias are often met with in the progeny of drunkards.

Drs. Shuttleworth and Beach traced a family history of insanity or imbecility in 21·38 per cent. of their cases of mental defect in children, and as great reticence is usually observed in the acknowledgment of such a taint, it may be safely assumed that the real proportion was higher. Dr. Kerlin states that 28 per cent. of his imbecile children were of insane or weak-minded stock; and there is a consensus of opinion that at least 25 per cent. of all idiots are epileptic.

* Trans. Path. Socy., 1869.

* Hack Tuke's Dicty. Psych. Med., p. 664.

† Strahan, "Marriage and Insanity," p. 126.

---

In connection with the Prince of Wales's Hospital Fund an official programme of the Royal Procession is to be issued; it is authorised by the Prince of Wales, and published on June 17th. It will contain sixty-four pages of letter-press description and illustrations, carefully drawn from life, of the different costumes and uniforms of those personages forming the Royal Procession on June 22nd. It will form a most useful guide to those able to be present, and an interesting memento of the day to be preserved by those unable to be there on the day. To secure copies an order should be given to newsagent, stationer, or at railway book-stall without a moment's delay. A space is left in each programme, where a specimen of the Prince of Wales's Hospital Stamp may be affixed, thus rendering the memento doubly interesting and valuable.

